# Research Overview on the Electromigration Reliability of SnBi Solder Alloy

**DOI:** 10.3390/ma17122848

**Published:** 2024-06-11

**Authors:** Wenjie Li, Liwei Guo, Dan Li, Zhi-Quan Liu

**Affiliations:** 1 School of Material Science and Chemical Engineering, Harbin University of Science and Technology, Harbin 150040, China; wj.li4@siat.ac.cn (W.L.);; 2 Shenzhen Institute of Advanced Technology, Chinese Academy of Sciences (CAS), Shenzhen 518055, China; 3 Shenzhen College of Advanced Technology, University of Chinese Academy of Sciences, Shenzhen 518055, China

**Keywords:** alloying element, electromigration, inorganic filler, reliability, SnBi

## Abstract

Due to the continuous miniaturization and high current-carrying demands in the field of integrated circuits, as well as the desire to save space and improve computational capabilities, there is a constant drive to reduce the size of integrated circuits. However, highly integrated circuits also bring about challenges such as high current density and excessive Joule heating, leading to a series of reliability issues caused by electromigration. Therefore, the service reliability of integrated circuits has always been a concern. Sn-based solders are widely recognized in the industry due to their availability, minimal technical issues during operation, and good compatibility with traditional solders. However, solders that are mostly Sn-based, such as SAC305 and SnZn, have a high melting point for sophisticated electronic circuits. When Bi is added, the melting point of the solder decreases but may also lead to problems related to electromigration reliability. This article reviews the general principles of electromigration in SnBi solder joints on Cu substrates with current flow, as well as the phenomena of whisker formation, voids/cracks, phase separation, and resistance increase caused by atomic migration due to electromigration. Furthermore, it explores methods to enhance the reliability of solder joint by additives including Fe, Ni, Ag, Zn, Co, RA (rare earth element), GNSs (graphene nanosheets), FNS (Fullerene) and Al_2_O_3_. Additionally, modifying the crystal orientation within the solder joint or introducing stress to the joint can also improve its reliability to some extent without changing the composition conditions. The corresponding mechanisms of reliability enhancement are also compared and discussed among the literature.

## 1. Introduction

In the process of solder production and usage, products containing Pb can cause environmental pollution. Therefore, in order to protect the environment and human health, multiple countries and regions have successively issued bans or restrictions on the use of products containing Pb [1]. Currently, as a solder widely applied as an alternative to Pb–Sn alloy in the electronic packaging industry, SnBi solder has been extensively utilized in microelectronics, automotive electronics, and the aerospace and telecommunications fields [2,3].

The melting point of SnBi solder is 139 °C [4], and it exhibits good wettability with metals such as Cu and Ni. As 3D IC devices continue to miniaturize, improve performance, and add more functionalities, SnBi solder faces a significant increase in current density in single solder joints in electronic packaging [5]. This results in higher temperatures and stresses within the solder joint. In chip applications, the operating temperature range for high-reliability integrated circuits is −25 to 125 °C [6]. Additionally, due to electrical reasons, there are stresses between the substrate and solder joint, as well as within the solder joint caused by electron wind. Fortunately, SnBi solder has a low melting point, high solderability, and excellent wetting properties. It can quickly fill gaps and form uniform solder joints during the soldering process, effectively addressing the issues that arise. However, SnBi solder also has some drawbacks. It exhibits relatively poor physical properties making it prone to defects such as voids and cracks, which can affect the reliability of solder joints. SnBi solder has low mechanical strength, making it susceptible to external forces and vibrations, leading to phenomena such as electromigration and thermal fatigue that can cause circuit failure [7]. It is necessary to take effective measures to mitigate the impact of these issues. This paper provides an overview of the electromigration phenomenon of SnBi solder in the packaging field and discusses methods to alleviate the problems caused by electromigration. Hopefully, this will provide some valuable insights for future packaging technologies.

## 2. Electromigration Theory

Electromigration is the phenomenon of migration and diffusion of metal atoms under the action of a large current [8,9].

Under the influence of an electric field, the metal within a weld joint gains sufficient energy from the externally applied current, causing outer-shell electrons to dissociate from their atoms. These liberated free electrons, accelerated by the electric field, acquire kinetic energy and collide with ions present in the weld joint. The kinetic energy acquired by the electrons is then transferred to the metal particles within the weld joint. This energy transfer induces movement of the metal particles.

**Figure 1:**
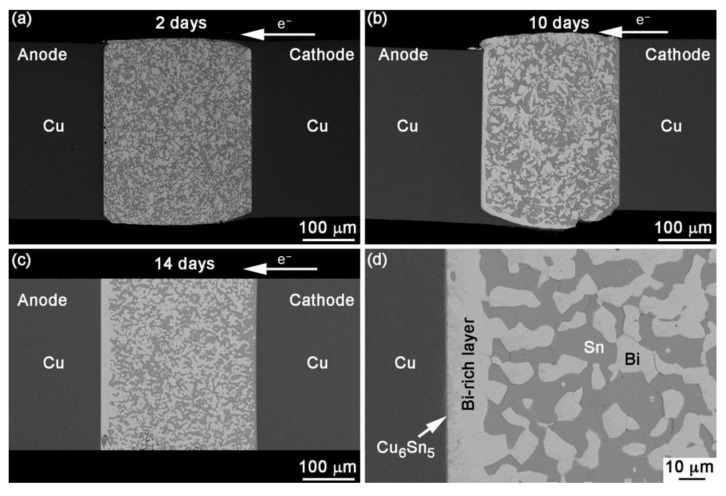
BSE images showing the sequence of events after EM with current density of 1.0 × 10^4^ A/cm^2^ at 30 °C for (**a**) 2 days, (**b**) 10 days, (**c**) 14 days and (**d**) anode interface in a joint stressed for 14 days [12].

As the metal particles move, the Cu_6_Sn_5_ and various components within the weld joint becomes non-uniform, resulting in gradients, as show in Figure 1. Concurrently, the impact of the electrons also gives rise to stress within the material, known as electron impact stress [10].

It is worth noting that the process is also influenced by electron wind forces, contributing to stress within the material. These interactions involving electron energy transfer and collisions play a crucial role in the formation and development of weld joint, influencing the microstructure and performance characteristics of the welded region.

In conclusion, the electric migration flux J is composed of flux induced by electron wind ($J_{em}$) [11], migration flux ($J_{chem}$) caused by chemical gradients, and flux of back-stressed atoms caused by stress ($J_{\sigma }$), and can be expressed as the following [12]:

(1) $$J=J_{\sigma }+J_{chem}+J_{em}$$

(2) $$J=-C\Omega \frac{D}{kT}\frac{\partial \sigma }{\partial x}-D\frac{\partial C}{\partial x}+C\frac{D}{kT}Z^{*}e\rho j$$

where Z* is the effective charge constant of electromigration; *C* is the concentration; *D* is the diffusion coefficient; *x* is the displacement distance; *T* is the absolute temperature; *∂σ*/*∂x* is the stress gradient; *ρ* is a resistance; *j* is the current density; *k* is the Boltzmann constant; Ω is the atomic volume; and *e* is the charge of the electron.

**Figure 2:**
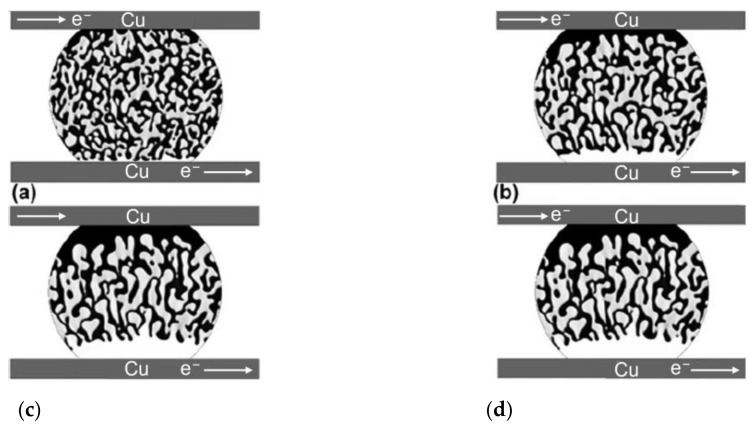
The process of electromigration morphological evolution of the microstructure of Sn58Bi solder in a flip chip Cu/Sn58Bi/Cu joint under electric current stressing for different nondimensional times: (**a**) τ = 10, (**b**) τ = 50, (**c**) τ = 100, and (**d**) τ = 150 [14].

During the electromigration of SnBi solder, a phase separation occurs, leading to the formation of two distinct phases and the occurrence of open circuits simultaneously. In the initial stage, under low current density and low temperature conditions, due to the higher carrier concentration of Bi atoms compared to Sn atoms, Bi atoms diffuse faster than Sn atoms [13]. As shown in Figure 2, the atomic flux caused by electron wind contributes significantly to the overall flux J, where $J_{em}$ serves as the main driving force for the directed migration of Bi atoms to the anode [14]. At the cathode interface, tensile stress is generated due to the loss of Bi atoms. Simultaneously, in the presence of Bi reaching the anode, a stress gradient is formed within the solder joint from cathode to anode. Driven by this compressive stress, Sn atoms migrate from the anode to the cathode, forming $J_{\sigma }$. It is worth mentioning that compound Cu_6_Sn_5_ can be observed on the surfaces of both the anode and cathode Cu substrates, and there is a possibility of Cu_3_Sn formation [15]. As the electromigration progresses, Kirkendall voids appear in the solder joint, resulting in the formation of an electric bridge effect. The current density increases, leading to a rapid increase in Joule heating within the solder joint. The molten Cu from the cathode enters the solder and migrates towards the anode in the direction of the current. At this stage, the primary migrating elements become Sn and Cu, with $J_{chem}$ and $J_{\sigma }$ surpassing $J_{em}$. Therefore, $J_{chem}$ becomes the main driving force [14,16]. Consequently, Bi atoms undergo reverse diffusion into the solder matrix. Finally, a complete phase separation occurs or an open circuit takes place within the solder joint. As shown in Figure 2c, Bi atoms continuously diffuse to the anode and accumulate, resulting in the formation of a continuous Bi layer on the anode. Conversely, a continuous Sn-rich layer forms on the cathode [17,18].

## 3. Defects Induced by Electromigration of SnBi Alloy on Cu Matrix

### 3.1. General Electromigration Behavior of SnBi Alloy Concerning Its Crystal Structure

During electromigration, atomic migration occurs, accompanied by an increase in atomic activity due to elevated temperatures, resulting in morphological changes at both the anode and cathode of the solder. Small mounds form at the anode, while depressions form at the cathode. Additionally, Sn–Bi hybrid whiskers are formed at the anode, while large cracks appear at the cathode.

**Figure 3:**
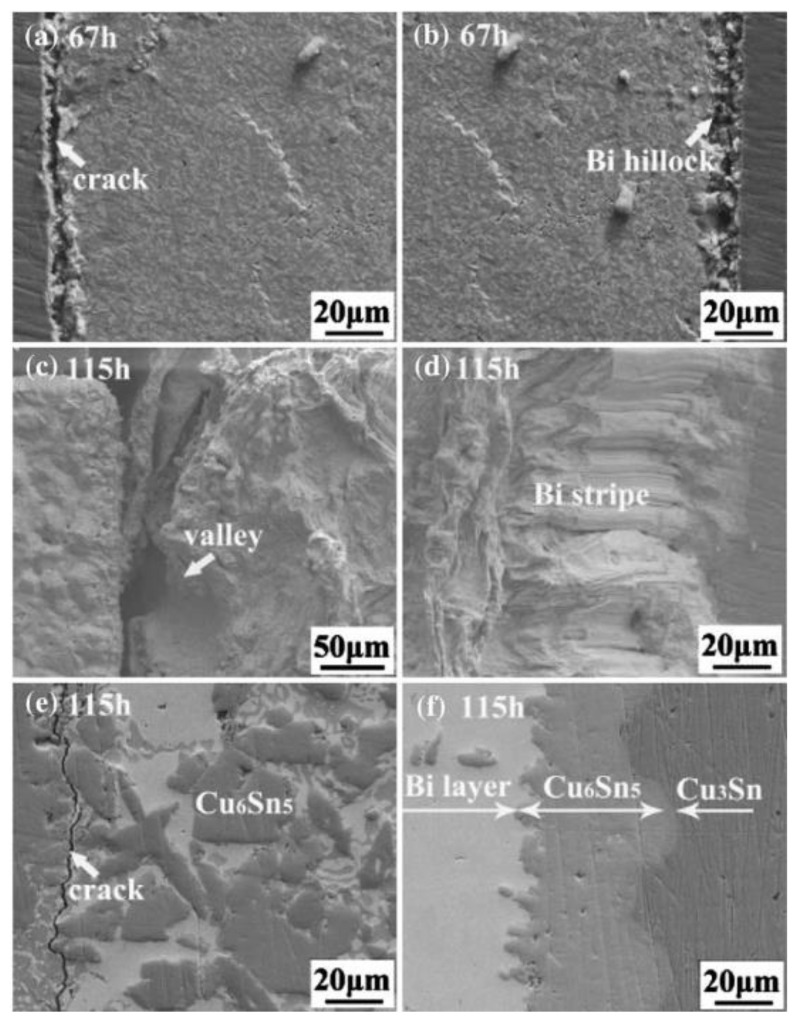
SEM images of SnBi with current density of 5 × 10^3^ A/cm^2^ under high temperature of 100 °C (**a**,**c**,**e**) at the cathode, and (**b**,**d**,**f**) at the anode [32].

Within the solder, there are compressive stresses induced by electron wind force in the direction of the electron flow, and there are tensile stresses extending outward in the direction perpendicular to it. At the same time, the growth of block-like Cu_6_Sn_5_ (IMC) results in an increased volume for the mixed phase of Sn and Bi on top [19]. Bi has a rhombohedral crystal structure, while Sn has a body-centered tetragonal structure. The rhombohedral crystal structure is less prone to exhibit plastic deformation through slip, so stress relief first occurs in the Sn portion at the anode, but the arrival of Cu consumes Sn atoms. Particles of Bi elements mix with Cu_6_Sn_5_, inhibiting excessive grain growth. Under these conditions, as shown in Figure 3b below [20], at the anode interface, a Bi-enriched structure squeezes out from the surface, forming a continuous row of hills consisting of a mixture of Bi and Sn, with Bi being the predominant component accompanied by a small amount of Sn [21]. Additionally, as more Bi and Cu atoms accumulate at the anode, the stress causes the mixed whiskers to be squeezed out to the surface [22,23].

During the process of electromigration, the Cu substrate dissolves into the solder due to excessive current density, and the primary migrating elements become Sn and Cu [12]. As Bi atoms continuously leave the cathode region [24], they create tensile stress at the interface between the solder joint and the substrate [25]. The reaction between Cu and Sn produces Cu_6_Sn_5_, leading to a chemical gradient in the solder with Cu atoms, which act as the reactant, migrating from the cathode to the anode [26]. Under the influence of electron wind force and chemical potential gradient, Cu atoms continuously migrate from the cathode to the anode, causing the local Cu concentration near the cathode to drop below saturation and resulting in continuous melting of the Cu substrate [27]. As a result, Bi and Cu all migrate towards the anode, leading to significant consumption of the solder near the cathode surface and the formation of uneven depressions. The extensive void splitting at the cathode interface is shown in SEM image Figure 3e below.

Another prominent phenomenon in the microstructural evolution of the Sn–58Bi solder joint during electromigration is the coarsening of the Bi phase [12]. Similar coarsening phenomena have been observed in the Pb phase of Sn-Pb solder and the Ag_3_Sn phase of Sn-Ag-Cu solder during electromigration [28,29].

The coarsening of the microstructure in the solder joint can be attributed to two main factors. In the eutectic solder system of Sn–58Bi, the driving force for Bi coarsening is the reduction of specific interfacial energy at grain boundaries or Sn–Bi interfaces [30]. At lower current densities, the growth mechanism of the Bi phase is primarily controlled by dislocations. As the current density increases, diffusion in the Bi phase transitions from being controlled by dislocations to being controlled by volume or interfaces. The activation energy required for diffusion via dislocations or grain boundaries is lower than that for volume diffusion or growth controlled by interfaces.

Firstly, under the influence of applied temperature, the coarsening of the Bi phase is mainly due to slow atomic migration or motion of grain boundaries. As the temperature increases, diffusion along grain boundaries synergistically interacts with diffusion via dislocations. Secondly, with increasing current density, the application of current on the Sn–58Bi solder joint not only promotes an increase in solder temperature due to Joule heating but also enhances the density of collisions between electrons and atoms. Consequently, the dominant diffusion mechanism shifts towards volume diffusion or even interface-controlled growth [31].

### 3.2. Electromigration-Induced Crack Formation

The atomic migration induced by SnBi solder electromigration results in the formation of stress and Kirkendall voids due to compositional inhomogeneity. These factors contribute to the generation of crack sources in solder joints, which rapidly nucleate and grow. Under high-temperature conditions, the solder joint experiences creep, intensifying the rapid growth of cracks, ultimately leading to complete open circuitry of the solder joint.

**Figure 4:**
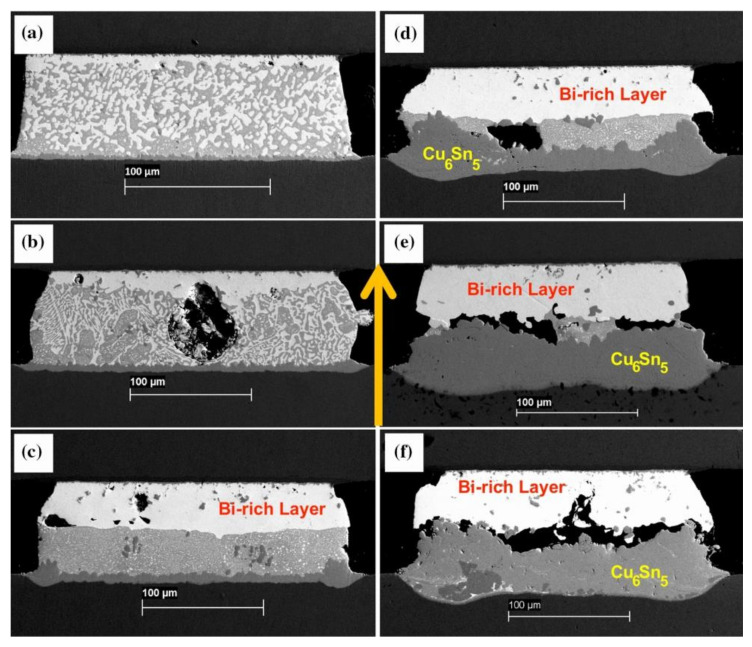
Scanning electron micrographs of Cu/Sn57BiSbNi/Ni solder joints which have been current-stressed for (**a**) 100 h, (**b**) 200 h, (**c**) 400 h, (**d**) 800 h, (**e**) 2200 h, and (**f**) 2500 h at a temperature of 125 °C and a current density of 4 kA/cm^2^. The yellow arrow indicates the electron current direction (down) (color figure online) [36].

The main cause of crack initiation from Bi enrichment is the migration of Bi from its original position towards the anode, leaving voids in its place [33]. Liu et al. [34] employed a current density of 1 × 10^4^ A/cm^2^ on a weld with a cross-sectional area of 5 × 10^−4^ cm^2^ for an electromigration investigation spanning 25 days. They documented the development of cracks in the SnBi layer at intervals of 10 days, 15 days, 20 days, and 25 days, respectively. Bi itself has a high electrical conductivity and thermal resistance but low thermal conductivity, leading to a higher temperature on the anode side of the Bi-enriched region than the cathode side. The adhesion between the Cu substrate and the Bi-enriched layer is weak. At different temperature stages, cracks exhibit different effects. Cracks nucleate in the weld joint at low temperatures, with atomic movement direction migrating towards the region of stress concentration [35,36]. As depicted in Figure 4, the weld underwent electromigration for 2500 h at a current density of 4 kA/cm² and a temperature of 125 °C, leading to significant cracking of the weld. In the case of continuous current flow, the electric bridge effect occurs in the weld joint, leading to a temperature rise after electric migration. The difference in coefficient of thermal expansion between the anode Bi-enriched region and the cathode causes additional damage under stress and provides more nucleation sites for cracks. Expansion generates internal stress that promotes the aggregation and diffusion of cathode voids, ultimately resulting in an interface fracture.

On the other hand, with increasing stress, temperature, and current density, the steady-state creep rate of the weld joint significantly increases, indicating a more severe electro-thermo-mechanical coupling load that accelerates the failure of the weld joint [37]. This is attributed to enhanced overall and localized Joule heating, as well as strain mismatch at phase interfaces. The growth of voids, due to defects in the material, leads to the breakdown of the weld joint, reducing the resistance to deformation and promoting creep deformation. Atomic migration induced by electromigration accelerates the proliferation and movement of dislocations, where strong electron wind forces disrupt the lattice structure, rapidly increasing the dislocation density.

In general, the cause of weld joint cracking is the result of the coupling effect of electrical current stress and elevated temperature.

### 3.3. Phase Separation Phenomenon Caused by Electromigration

During the electromigration process, phase separation occurs as a result of the main migrating atoms being driven towards the anode by the electron wind force under the influence of electric current. In the solder joint of SnBi solder, the initially homogeneous SnBi alloy transforms into two distinct phases, with Sn and Bi occupying the cathodic and anodic ends respectively [38].

The direct cause of the formation of phase separation is the acceleration and diffusion of atoms in the direction of electron flow due to electronic wind [39]. The magnitude of the electronic wind depends on the current density, and the current density determines the main migrating substance.

**Figure 5:**
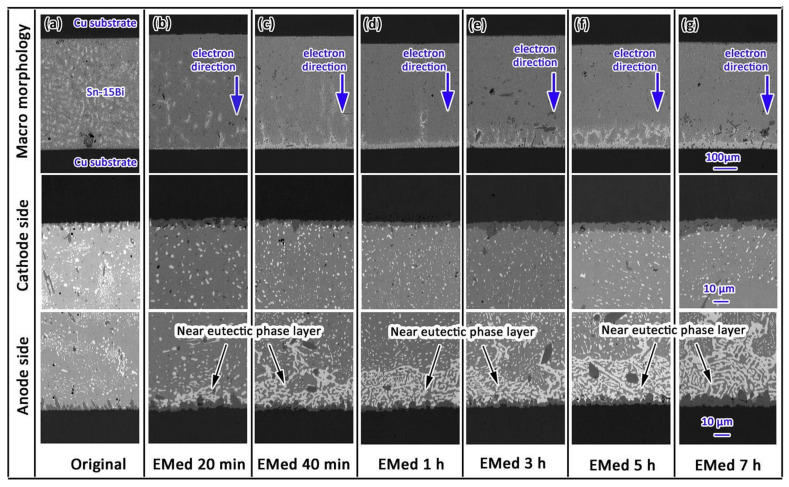
Macro morphology, cathode side morphology and anode side morphology of original and electro-migrated Sn-15Bi solder joint. (**a**) Original, (**b**) electro-migrated for 20 min, (**c**) electro-migrated for 40 min, (**d**) electro-migrated for 1 h, (**e**) electro-migrated for 3 h, (**f**) electro-migrated for 5 h, and (**g**) electro-migrated for 7 h [42].

Under low electrical current density, Bi migrates from the cathode to the anode under the influence of electron wind force and electrostatic field. Due to the abundance of charge carriers in Bi atoms compared to Sn atoms, Bi diffusion is faster than that of Sn. Thus, a large number of Bi atoms accumulate at the anode interface, generating compressive stress [40,41]. At the cathode interface, the loss of Bi atoms produces tensile stress, creating a stress gradient from cathode to anode inside the joint. Driven by stress, Sn atoms then migrate from anode to cathode. Over time, Bi atoms continue to diffuse towards the anode and accumulate, eventually forming a continuous Bi layer. Since Bi has a higher electrical resistance, the energizing of the joint generates a significant amount of Joule heat in the Bi layer at the anode interface, leading to a temperature gradient between the anode and cathode [42], further promoting Bi atom diffusion and migration, resulting in thickening of the Bi layer. In Figure 5, it is demonstrated that at a temperature of 150 °C and a current density of 160 A/cm^2^, electromigration takes place without the presence of a eutectic component in the weld. Following the onset of electromigration at 20 min, there is a shift in the atom composition due to the influence of electron movement. Over time, the near-eutectic layer’s composition within the weld expands from 0 μm to 34.68 μm over a period of 7 h.

Under high current density conditions, the main migrating substances are Cu and Sn. In a system composed of these two components, atomic migration depends on the magnitude of the current. Bi atoms have more outer electrons and charge carriers compared to Sn atoms [35]. Therefore, as the dominant atoms, Bi atoms migrate towards the anode, forming an enrichment layer at the anode under low current density conditions [12]. However, at higher current densities, the Sn–58Bi solder alloy liquefies through Joule heating, promoting the dissolution of Cu atoms from the substrate into the molten solder [11]. At lower current densities, where Cu atoms do not dissolve into the solid Sn–58Bi solder and the chemical gradient is almost negligible, the higher atomic flux of Bi atoms compared to Sn atoms becomes the main migrating element.

Meanwhile, temperature changes affect the rate of atomic diffusion [43]. At high temperatures, the increased thermal motion and collision frequency between atoms accelerate the diffusion of Bi atoms in the SnBi joint. In contrast, at lower temperatures, diffusion requires higher current densities to activate atomic migration. The higher the stress temperature, the higher the current density, and the longer the stress time, the thicker the enriched Bi layer becomes. Zhang et al. [44] found that under the action of L-SEM, Bi atoms migrate from the cathode to the anode during the heating stage. At 140 °C, a three-layer equilibrium is maintained, while at the insulation stage at 170 °C, an approximately uniform phase is obtained. Bi atoms continue to migrate from the cathode to the anode until the cooling stage, where the enriched Sn phase and enriched Bi phase are completely separated.

### 3.4. Effect of Electromigration on Resistance

During the process of electromigration, phase separation and void formation lead to a rapid increase in resistance within the SnBi solder joint as the current is applied over time. Eventually, this results in an open circuit due to the significantly elevated resistance.

**Figure 6:**
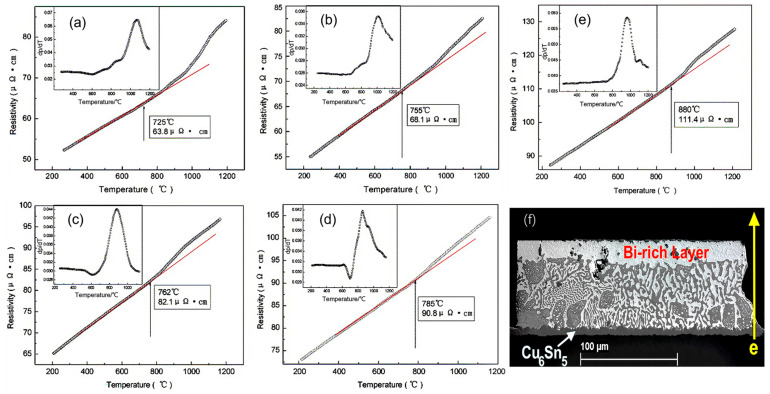
The resistivity–temperature and their coefficient curves of liquid Sn-Bi alloys during heating: (**a**) SnBi20; (**b**) SnBi30; (**c**) SnBi40; (**d**) SnBi57; and (**e**) SnBi80. (**f**) Microstructure of Ni/Sn57Bi0.5SB0.01Ni/Cu solder joints 200 h under current stress of 4 kA/cm^2^ at 125 °C [45,50].

During the electromigration process, the resistance changes as atoms undergo movement. As the temperature of the solder joint increases, the resistivity of the entire joint also increases [45,46]. In Figure 6, it is evident that variations in the SnBi composition ratio led to changes in resistance. Specifically, as the proportion of Bi increases from 20% to 80%, there is a substantial increase in resistance at the same temperature. Consequently, the region where resistance heat is generated during the electromigration process becomes non-uniform. The elevated proportion of Bi components near the anode end results in increased resistance heat, thereby accelerating the movement of atoms during electromigration. In the field of microelectronics, resistance represents the lifetime of components, and a 7% increase in resistance indicates the failure of the solder joint connection. ZUO et al. discovered three stages of resistance change in SnBi solder joints caused by electromigration when studying the thermoelectric coupling effect of SnBi solder [47]. In the phase coarsening stage, the resistance decreases due to the increase in grain boundary energy. In the second stage, as the temperature rises, the resistance begins to slowly increase. In the final stage, the combined effect of thermal cycling and phase separation leads to a rapid increase in resistance. Similarly, SUN et al. [48] also discovered three stages of resistance increase during SnBi electromigration research. After 600 h of 5 A current stress, the resistance of the solder joint remains stable without significant changes in the first stage. In stage II, after 120 h of current stress at 25 °C, a Bi-rich layer forms on the anode side. This Bi-rich layer can be defined as an independent resistor with a higher resistivity, which further promotes voltage increase. Therefore, the Bi-rich layer formed at the solder joint interface causes resistance in stage II of the solder joint. At the same time, the thickness of the Bi-rich layer linearly increases over time until the different phases are completely separated. Finally, after 600 h of current stress, phase separation is complete, and the resistance rapidly increases in stage III, leading to complete failure of the solder joint.

The resistivity of the Bi phase is greater than the sum of the resistivity of the remaining Sn, Cu, and eutectic SnBi phases. Atomic migration causes redistribution of the phases in the solder joint, and the enrichment of the Bi phase leads to a rapid increase in resistance [49]. The different potentials between the Bi-rich phase and Sn-rich phase during electromigration results in the formation of voltage, and the voltage in the solder joint increases with the degree of atomic migration. After complete electromigration, the resistance of the solder joint stabilizes at a certain value, indicating an increased resistance [50]. Moreover, the conductivity of Bi differs from other components, resulting in non-uniform distribution of current density in the solder joint. Due to the higher resistivity of Bi, the current density in the Bi-rich phase is lower compared to other phases [34]. Thus, the resistance is caused by the different resistivity values of the scattered intermetallic compounds and enriched phases formed in the solder material after electromigration, leading to disrupted electron flow and increased resistance. After electromigration is completed and the Bi has migrated towards the anode, forming a thick Bi-rich layer, the resistance also increases as the grain coarsens. The resistance changes accordingly with different SnBi compositions, increasing with the increase in Bi content. In an experiment conducted by Murayama et al. [51], it was found that the maximum resistance increase in the Sn–Bi eutectic solder was 82%, accompanied by the formation of a thick Bi layer under current stress. Using Sn30Bi solder, the maximum resistance increase after current stress was 20%.

**Figure 7:**
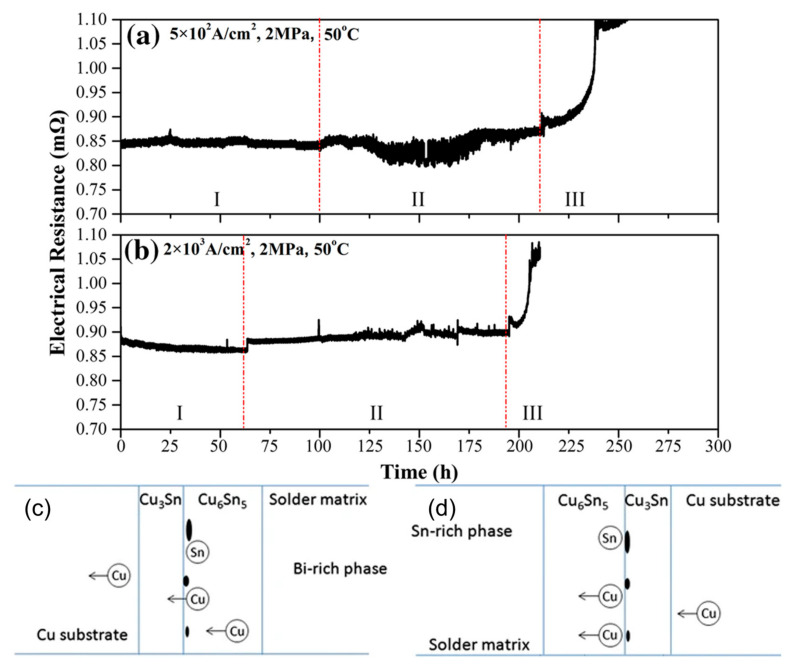
Electrical resistance changes under the conditions: (**a**) 5 × 10^2^ A/cm^2^, 2 MPa; (**b**) 2 × 10^3^ A/cm^2^, 2 MPa. Schematic drawing of formation of IMCs and voids: (**c**) the anode side; (**d**) the cathode side [48,52].

In the process of low-temperature thermal cycling, the main cause of resistance increase is the change in microstructure, which becomes increasingly significant under continuous current flow [52]. Cu_3_Sn is continuously distributed between Cu_6_Sn_5_ and the Cu substrate. The stable Cu_3_Sn layer impedes the movement of Sn atoms towards the Cu substrate. Under the influence of electron wind force, Cu atoms from Cu_6_Sn_5_ migrate to the Cu_6_Sn_5_–Cu_3_Sn interface, where they synthesize Cu_3_Sn with Sn. This leads to an increase in the required ratio of Cu atoms to synthesize Cu_3_Sn, resulting in localized insufficient Cu atoms and the formation of Kirkendall voids. Under conditions of increased current and temperature, the decomposition of Cu_6_Sn_5_ into Cu_3_Sn is promoted, releasing Sn atoms from the Cu_6_Sn_5_. Additionally, it is worth noting that Sn and Cu can also form Cu_3_Sn, as shown in Figure 7 [48,52]. The formation and propagation of cracks lead to a reduction in the cross-sectional area of the solder joint, increasing the open circuit resistance [53]. Under thermal cycling, the voids begin to expand and merge, and cracks nucleate and propagate, ultimately resulting in open circuits and complete failure of the solder joint.

## 4. Methods and Measures of Electromigration Mitigation for SnBi Alloy on Cu Substrate

### 4.1. Addition of Alloying Element into SnBi Solder

#### 4.1.1. Effect of Fe Addition on the Properties of the Solder

In order to prolong the lifespan of solder joints subjected to electromigration, various scholars have proposed numerous approaches. One such method is the addition of Fe particles, which effectively suppresses the growth of Cu_6_Sn_5_ (IMC) and refines the solder microstructure, thereby enhancing the reliability of the solder joint [54].

By adding Fe particles into SnBi solder joints, most of the Fe will precipitate as FeSn_2_ phase or in coexisting regions due to its low solubility in Sn. Some Fe will dissolve into Cu_6_Sn_5_ and substitute for Cu positions. The coexisting regions may also potentially form oxides.

Fe_2_O_3_ is dispersed in the solder joint [55], inhibiting the growth of IMC and providing nucleation sites for refined grain structure, thereby improving the mechanical performance of the solder joint. Oxides are non-conductive and do not undergo movement due to electron wind, allowing them to withstand the kinetic energy brought by Bi and Cu, thus mitigating atomic migration caused by electron wind.

**Figure 8:**
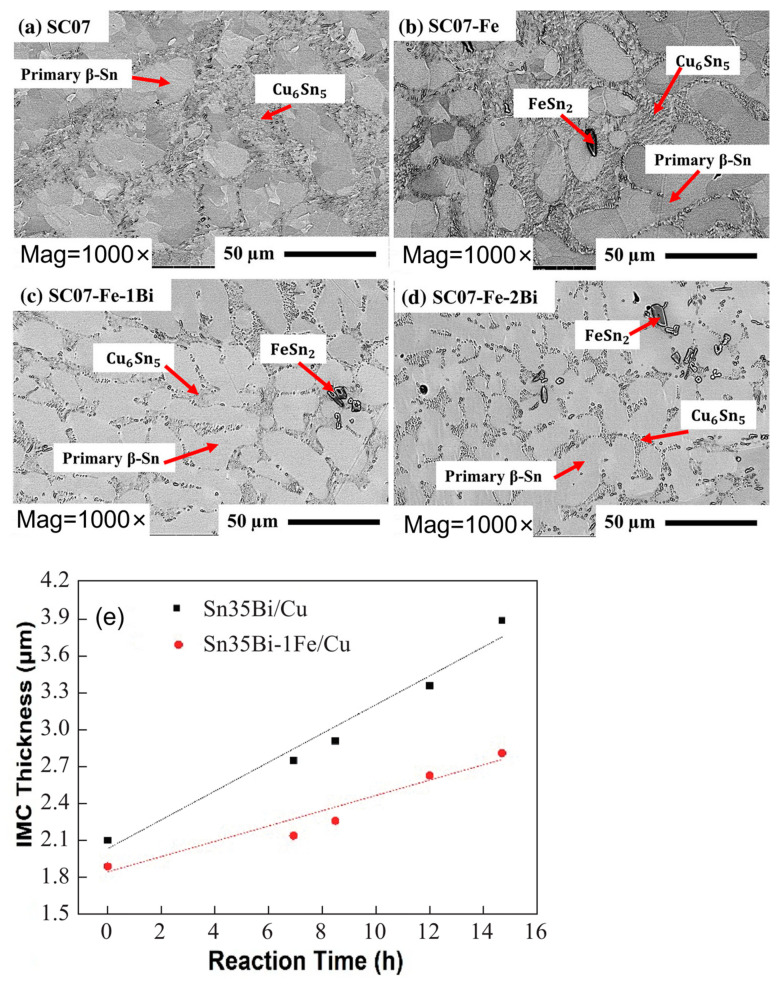
FESEM micrographs of as-cast (**a**) SC07, (**b**) SC07-Fe, (**c**) SC07-Fe-1Bi, and (**d**) SC07-Fe-2Bi solder alloys. And (**e**) diagram of the relationship between the thickness and aging time of the interfacial IMC layer of Sn35Bi-1Fe/Cu and Sn35Bi/Cu at constant temperature aging [57,60].

In addition to oxide formation, Fe reacts with Sn in the solder to form FeSn_2_, which is dispersed in both the solder matrix and the IMC layer, inhibiting electromigration [56,57]. At the same time, FeSn_2_ partially substitutes Cu_6_Sn_5_ in the solder joint [58]. Fe can dissolve into Cu_6_Sn_5_ [59], and its substitution for Cu introduces a lattice misfit of 0.31%. This results in an increase in lattice strain in Cu_6_Sn_5_, reducing the vacancy diffusion rate and slowing down grain coarsening in the solder joint [60]. As demonstrated in Figure 8, FeSn_2_ is dispersed in the interdendritic region and does not alter the overall weld structure. Nonetheless, as the aging process occurs, the IMC within the weld diminishes swiftly due to the substitution of Fe for Cu in Cu_6_Sn_5_.

Li et al. conducted electromigration experiments on composite solders made by adding Fe particles into SnBi solder [61] under a current density of 0.6 × 10^4^ A/cm^2^. In the experiment, the reference group without Fe particles exhibited the morphology reported in the previous literature, with white substances accumulating at the poles after prolonged current flow. In contrast, the composite solder group with added Fe particles showed a decelerated growth of IMC during electrical stressing, as the metallic compounds resulting from the reaction between Fe and Sn hindered the formation of Cu_6_Sn_5_. It can be concluded from this that Fe particles can play a role in preventing the generation of electromigration defects.

#### 4.1.2. Effect of Ni Addition on the Properties of the Solder

One way in which Ni alleviates electromigration is through the formation of a compound (Ni,Cu)Sn_4_. This compound acts like a wall, restraining the migration of Bi. The compound particles are not sensitive to high currents and can serve as obstacles, impeding the rapid diffusion of phase boundaries in the eutectic SnBi system. As a result, the average velocity of Bi atoms/ions driven by electron wind significantly decreases, leading to an extended lifespan of solder joints [62].

Many researchers have found that the addition of Ni to solder can result in excellent mechanical performance and an improved operational lifespan at solder joints [56,63]. In their study on the role of Ni atoms in SnBi solder, Gu et al. [64] discovered that Ni influences the flow of current in the solder, impeding the movement of Cu. Additionally, in composite solders containing Ni, the atomic migration rate of Bi, driven by the electron wind force, is significantly reduced [62,65]. Overall, Ni has been observed to suppress preferential migration during electromigration within solder joints.

Ni forms multiple intermetallic compounds with Sn, including NiSn_4_, Ni_3_Sn, Ni_3_Sn_2_, and Ni_3_Sn_4_ [66]. However, in solder joints, Ni exists mainly in the form of a solid solution dissolved in Cu_6_Sn_5_ and as a compound that generates Ni_3_Sn_4_. Bashir et al. [67] studied the effect of Ni and Ni-P on electromigration in Sn-based solders and found that under equilibrium cooling conditions, Ni primarily forms Ni_3_Sn_4_ compound, leading to low strength and brittle fracture at the joint. In the study by Chen et al. [68] on Ni/Au metallization of SnBi under electromigration, it was found that Ni mainly forms Ni_3_Sn_4_ in the growing compound, and long-term current flow results in severe phase separation at both ends. Clearly, Ni_3_Sn_4_ is not the desired compound in the electromigration process.

The formation of (Cu,Ni)_6_Sn_5_ occurs under non-equilibrium cooling conditions due to the cubic crystal structure of Ni atoms, which is similar to the crystal structure of Cu atoms [69]. Xu et al. [70] have found that Cu_6_Sn_5_ and (Ni_1−x_Cu_x_)_6_Sn_5_ have highly similar structures, confirming the structural similarity of the newly formed intermetallic compounds. Additionally, Ni has a higher affinity for Sn compared to Cu [71]. Therefore, Ni atoms partially replace Cu atoms in Cu_6_Sn_5_, leading to the formation of (Cu,Ni)_6_Sn_5_ at the interface and within the solder. The formation of (Cu,Ni)_6_Sn_5_ reduces the dissolution of Cu and hampers the flux of Cu atoms from the cathodic side of the interface to the anodic side, thereby reducing the defects caused by polarity effects [72]. Furthermore, the addition of Ni inhibits the growth of Cu_3_Sn and reduces void formation [36], thereby improving the mechanical properties of the solder joint. The activation energy for the coarsening of (Cu,Ni)_6_Sn_5_ is much larger than that for the rich Bi phase, making it stable at low temperatures. In strength experiments conducted by Mao et al. [73] with the addition of foamed Ni into SAC305, it was found that the shear strength of the (Cu,Ni)_6_Sn_5_ compound in the non-electrified condition reached 76.89 MPa. However, under excessive current conditions, the strength decreased to 21.65 MPa compared to the original strength.

**Figure 9:**
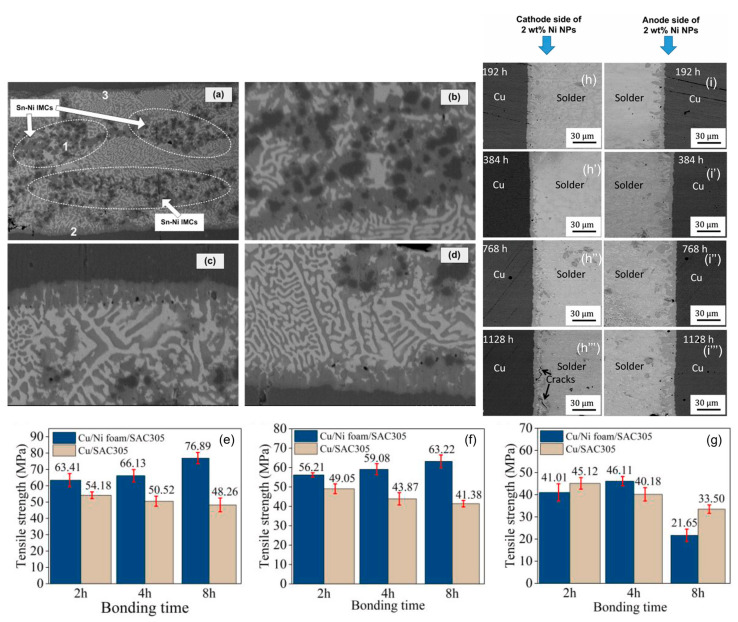
As-reflowed microstructure of Ni-particles reinforced composite solder joint: (**a**) full view of the solder joint; (**b**) enlarged micrograph of region 1 from (**a**); (**c**) enlarged micrograph of region 2 from (**a**); and (**d**) enlarged micrograph of region 3 from (**a**). Tensile strength of Cu joints under the condition of 320 °C for 2, 4 and 8 h with the electric densities of (**e**) 0 A/cm^2^, (**f**) 3.33 × 10^2^ A/cm^2^, and (**g**) 6.67 × 10^2^ A/cm^2^. FESEM backscattered electron images of SAC305 + 2 wt% Ni NPssolder joint after the EM test for 192, 384, 768 and 1128 h. (**h**–**h′″**) show the cathode side, and (**i**–**i′″**) show the anode side [39,73,74].

The Figure 9 shows the results of an electric migration experiment conducted by Bashir et al. [39] with the addition of 2% Ni nanoparticles in the solder. After a long period of electrification, the Cu_6_Sn_5_ (IMC) in the reference group showed a scallop shape, while the IMC in the composite solder group remained flat without excessive growth, proving that Ni suppresses the polarity effect and reduces cracks, voids, and damages in the solder, thereby lowering the average anode growth rate of IMC [74]. It can reasonably be concluded that the addition of Ni doping reduces electromagnetic damage and is expected to prolong the service life of the solder joint.

#### 4.1.3. Effect of Ag Addition on the Properties of the Solder

The addition of Ag has a mitigating effect on electromigration in the SnBi solder [75]. Ag is present in the form of dispersed Ag_3_Sn at the SnBi phase boundary, which enhances the strength of the solder joint [40,76]. The incorporation of Ag particles serves to increase the reliability during the electromigration process by impeding atomic migration through the formation of the Ag_3_Sn compound with Sn, thus prolonging the operational lifespan of SnBi solder joints [77].

**Figure 10:**
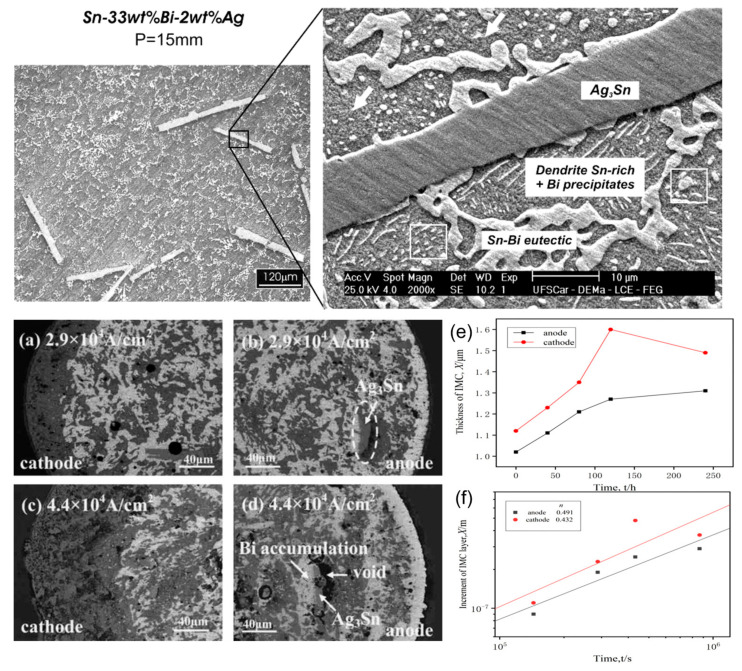
SEM and optical images of transverse sections detailing the microstructures Sn–33 wt.%Bi–2 wt.%Ag alloy. (**a**–**d**) current 2.9 × 10^4^ A/cm^2^ and 4.4 × 10^4^ A/cm^2^ role after 360 h Ag doping solder strip internal microstructure. Interface IMC layer thickness with power-on–time curve (**e**) Cu/Sn–58Bi–1Ag/Cu. (**f**) Cu/Sn–58Bi/Cu [80,83,87].

From a morphological perspective, the Ag_3_Sn particles vary in shape depending on the cooling state and Ag-Sn content. Hsu et al. [78] studied the interface reaction of Sn-Co/Ag and Sn-Co/Cu under electrification, and found that needle-like Ag_3_Sn particles precipitate and grow at equilibrium, while spherical Ag_3_Sn rapidly solidifies upon quick cooling. It can be determined that Ag_3_Sn, formed by the reaction of Ag and Sn in the solder layer, is stable [79,80]. Ma et al. [81] investigated the effect of Co addition in Sn-3.0Ag-0.5Cu-based solder joints on electromigration behavior and found that Ag_3_Sn is distributed within the solder joint, and even after 3 days of continuous electrification, Ag_3_Sn particles remain in a dispersed state without atomic migration. After long-term electrification, the presence of Ag_3_Sn exhibits an intercepting effect, with small Bi-enriched regions appearing on one side and depletion on the other side, resulting in a reduction in the thickness of the Bi-enriched region on the anode side [82]. In order to study the effect of Ag electromigration in SnBi solder, Chen et al. [83] investigated the morphology of the entire solder joint and the distribution of each component under different current densities. Figure 10 shows the microstructure of Sn-Bi-Ag alloy under two different current density conditions. Ag_3_Sn blocks the atomic migration of Bi [84], but Bi in the region from the anode to the unblocked area is still affected by electron wind and migrates to the anode, forming a Bi-rich layer [85]. Zhang et al. [86], while studying the addition of Ag and Cu in SnBi creep, found that the addition of Ag hinders the dislocation motion of Ag_3_Sn, resulting in an increase in the creep stress exponent and a delay in phase separation in the solder joint. To investigate whether fine Ag_3_Sn can alleviate electromigration effects, Liu et al. [87] conducted a study using ultrasonic soldering to weld Ag composite SnBi joints. They found that fine Ag_3_Sn adsorbs at the phase boundary, effectively blocking the migration pathway for Bi atoms and inhibiting the formation of a Bi-rich layer. At the same time, the energy required for dislocation movement increases, enhancing the mechanical strength of the β-Sn phase.

However, the presence of Ag_3_Sn particles in SnBi solder is not without flaws. When Ag_3_Sn is unevenly distributed and aggregated in a region, its beneficial effect on electromigration in the solder joint decreases significantly. Additionally, Ag_3_Sn provides nucleation sites that are not uniform, accelerating crystal precipitation and refining grain structure in the solder joint, thereby providing numerous pathways for atomic migration. Smaller grain-sized Sn can obtain higher atomic flux, leading to increased formation of Cu_6_Sn_5_ and higher consumption of the Cu substrate, while the migration flux of Bi remains consistent with that before the addition of Ag. Sun et al. [88] found, in their study on the improved electromigration phenomenon of doped SnBi solder by Ag_3_Sn, that there is a thick layer of Cu_6_Sn_5_ adjacent to the anode and a Bi-rich layer.

On the other hand, the addition of Ag to Sn-based solder does have an impact on cathodic dissolution [89]. The influence of Ag addition on the consumption rate of the cathodic Cu substrate during electromigration is attributed to the decrease in activation energy caused by the introduction of silver. Lin et al. [42] investigated the effect of Ag addition on the consumption of the cathodic Cu pad in the Cu/Sn3.5Ag/Cu flip-chip solder structure under electromigration conditions. They conducted electromigration tests on Sn-based solder on a Cu substrate and found that, under the electromigration conditions, the activation energy for Cu dissolution into Sn-based solder is lower than that into Sn3.5Ag solder (1.48 eV). This is because, within a lower temperature range, the presence of Ag solute, which forms the Ag_3_Sn phase, adheres to the interface Cu-Sn compound layer and inhibits the dissolution of Cu_6_Sn_5_, thereby impeding the dissolution of the interface Cu_6_Sn_5_ compound layer [90].

In conclusion, the addition of the Ag element to SnBi solder can play a mitigating role in electromigration [83].

#### 4.1.4. T Effect of Zn Addition on the Properties of the Solder

Zinc (Zn) has a positive effect on mitigating electromigration. When added to SnBi solder in a compound formation manner, Zn can alleviate the formation of anode-side whisker protrusions and cathode-side voids during the process of electromigration. It also helps suppress the segregation of bismuth (Bi) at the anode interface and the growth of bismuth-rich layers.

Zn functions as an inhibitor of electromigration in a different way compared to other additives. Instead of forming a stationary barrier, it utilizes the back stress caused by electromigration to impede the migration of Bi atoms. At the same time, Zn atoms actively migrate from the anode to the cathode, filling the vacancies left by Bi atoms [91].

In the SnBi solder system, electromigration leads to the release of accumulated back stress, resulting in the migration of a large number of Bi atoms towards the anode region, forming a thicker Bi-rich layer [92]. In the presence of Zn in the solder joint, there is higher back stress during electromigration. Additionally, Zn at the anode can dissolve into the interfacial Cu_6_Sn_5_ (IMC) and form Cu_6_(Sn,Zn)_5_, which then diffuses into the Sn–58Bi matrix [93]. During electromigration, continuous growth of the anode interface IMC layer and dissolution of the cathode IMC layer hinders the formation of the Bi-rich layer [94].

**Figure 11:**
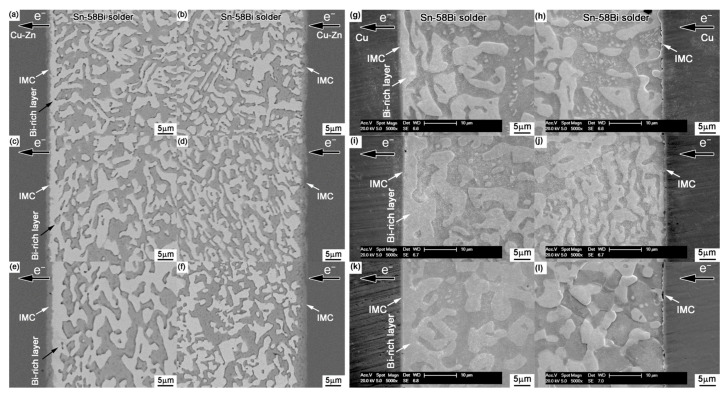
Interfacial structures in the Cu-Zn/Sn-58Bi/Cu-Zn solder joints after electromigration for various durations: (**a**,**b**) 33 h, (**c**,**d**) 66 h, and (**e**,**f**) 132 h. Interfacial structures in the Cu/Sn-58Bi/Cu solder joints after electromigration for various durations: (**g**,**h**) 33 h, (**i**,**j**) 66 h, and (**k**,**l**) 132 h [95].

**Figure 12:**
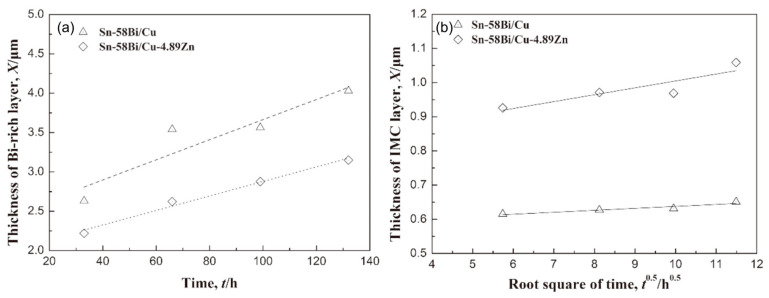
The thickness of the (**a**) Bi-rich layer and (**b**) IMC layer at the anode side with the stressing time [95].

In order to maximize the tensile stress in the SnBi system, some scholars directly alloy Zn on the surface of Cu and conduct electrical migration reliability verification. To study the addition of trace amounts of Zn alloying in the SnBi/Cu joint during electrical migration, Wang et al. [95] alloyed Zn on the surface of the Cu substrate, where the trace amounts of Zn in the substrate did not participate in the reaction between Cu and Sn. However, Zn also undergoes migration during electrical migration. Under the action of the current, Zn diffuses into the SnBi solder and IMC by diffusion into the solder joint. Figure 11 illustrates the comparison between electromigration aging with Zn alloying (left) and without alloying (right). The noticeable disparity lies in the thickness of the Bi-enriched layer. In the absence of Zn, it was observed that the Cu matrix dissolved, resulting in the formation of viods. Figure 12 further supports the observation that the presence or absence of Zn affects bismuth segregation in solder joints, and also leads to a slower growth rate in the Bi-rich layer as previously noted in Figure 11. Figure 12a illustrates that in the absence of Zn addition, the growth rate of the Bi-rich layer is faster with abnormal growth of the anode IMC. This is due to the enrichment of Bi, causing mutual diffusion of Cu and Sn in the anode substrate, leading to the formation of a thinner IMC layer compared to the Zn group.

#### 4.1.5. Effect of Co Addition on the Properties of the Solder

Cobalt (Co) is a relatively stable metal that is resistant to oxidation. It also possesses excellent magnetic properties and can be alloyed with other elements to manufacture high-strength magnets and wear-resistant tools. In solder joints, cobalt exists in the form of a solid solution and has a uniform distribution. Cobalt’s presence distorts the lattice structure, contributing to solid solution strengthening. It exhibits low distortion deformation and thermal stress at low temperatures. Additionally, cobalt plays a role in mitigating electromigration effects.

**Figure 13:**
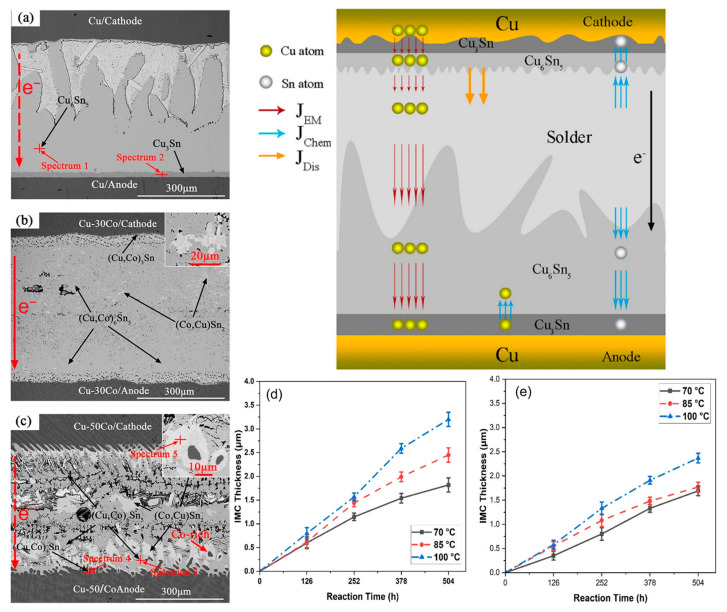
The cross-sectional SEM images and EPMA analysis of Cu-xCo/SAC305/Cu-xCo (x = 0, 30 and 50 wt.%) joints under the conditions of 260 °C, 2.89 × 10^2^ A/cm^2^ for 10 h, (**a**) Cu/SAC305; (**b**) Cu-30Co/SAC305; (**c**) Cu-50Co/SAC305 and IMC layer thicknesses; (**d**) Sn–Bi; and (**e**) Sn–Bi–0.5Co as a function of reaction time at various temperatures [96,97].

When Co is added to the SnBi system, it is not the Sn or Bi phases that undergo changes, but rather the Cu phase. Co atoms have the ability to enhance the stability of Cu_6_Sn_5_ and inhibit the growth of Cu_3_Sn [96]. The compound generated by Co in SnBi solder is (Cu,Co)_6_Sn_5_. Unlike Cu_6_Sn_5_ which aligns with the direction of the applied current, (Cu,Co)_6_Sn_5_ and the current are at a certain angle, improving the mechanical properties of the solder joint [97]. In the interfacial reaction, the binding energy between Co and Sn is greater than that between Cu and Sn. In Figure 13d,e, it is demonstrated that Co has a higher tendency to form compounds than Cu, as evidenced by the Cu_6_Sn_5_ (IMC) layer thickness being greater in samples without the Co addition compared to the experimental group with added Co during the aging period. Therefore, after melting, Co will precipitate and distribute uniformly in the (Cu,Co)_6_Sn_5_ matrix, providing numerous nucleation sites to form isolated (Co,Cu)Sn_2_ island-shaped intermetallic compound. Additionally, due to the precipitation and aggregation of Co, primary (Co,Cu)Sn_2_ grains disperse and nucleate around Co phases. These islands are not affected by the current and migrated material accumulates around them, highlighting the improved reliability of the formed compounds on the solder joint.

During electromigration, Co elements tend to aggregate at the center of the solder joint and distribute uniformly in a solid solution form, causing lattice distortion and enhancing mechanical strength [98].

#### 4.1.6. Effect of RE Element Addition on the Properties of the Solder

The addition of rare earth (RE) elements in the SnBi solder is commonly employed to improve the microstructure and properties of various alloys. Furthermore, the inclusion of rare earth elements partially alleviates electromigration. The presence of rare earth elements in SnBi solder reduces the interfacial energy and creates an energy difference, leading to grain refinement [99]. Due to the disparity in lattice energies between atoms within the bulk and at the interfaces, rare earth elements tend to migrate towards higher energy regions such as grain boundaries and interfaces, accumulating therein. As a result, dislocation and grain boundary sliding are hindered, making it more difficult for Sn and Bi atoms to migrate [100].

The addition of rare earth elements can lead to a decrease in interface energy and grain boundary size. The driving force for the accumulation of RE at the boundaries is the difference in lattice misfit energy between the interior of Sn/Bi atoms and the boundaries, which is caused by the presence of RE atoms [101]. The size difference between RE and Sn/Bi is significant. For example, the radius of Sn is 0.141 nm, while the radii of Ce and La are 0.183 nm and 0.187 nm, respectively, indicating that the rare earth atoms are larger than Sn atoms. Therefore, it is difficult for rare earth atoms to substitute for Sn atoms in the lattice [102]. In doped solders, the rare earth material forms a network enriched at the boundaries. This structure effectively restricts the migration of Sn and Bi atoms. The addition of rare earth elements leads to a decrease in conductivity, and the solid solution effect prolongs the duration of electromigration and diffusion, thus increasing resistance.

The characteristics of each rare earth element cannot be generalized. For instance, when the rare earth element Nd is added in large quantities, it forms compounds with Sn that promote the growth of Sn whiskers. Zeng et al. [103] conducted a study on the interface structure and properties of Sn-0.7Cu-0.05Ni/Cu solder joints with added Nd. They found that excessive addition of Nd to the Sn-based solder material resulted in the formation of a large number of Sn whiskers on the surface of the compound NdSn_3_. The significant generation of whiskers is driven by compressive stress, which is caused by the volumetric expansion resulting from the chemical reaction between the rare earth element and oxygen [104].

Furthermore, Y-element oxide can stabilize and exist in SnBi solder joints, similar to the function of Al_2_O_3_, inhibiting the growth of Cu_6_Sn_5_ (IMC) layers and refining the overall grain size in the solder joint. Liu et al. [105] investigated the impact of Y_2_O_3_ particles on the microstructure formation and shear performance of Sn–58Bi solder material and found that the shear strength of the solder material increased by 45% after the addition of Y_2_O_3_. However, the study of such oxides on electromigration reliability is not yet mature.

**Figure 14:**
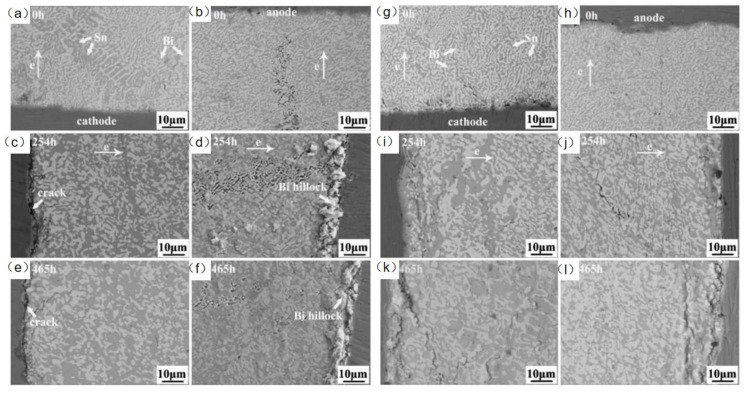
SEM images of Cu/Sn58Bi/Cu with current density of 5 × 10^3^ A/cm^2^ at room temperature (**a**,**c**,**e**) at the cathode, and (**b**,**d**,**f**) at the anode. SEM images of Cu/Sn58Bi0.1RE/Cu with a current density of 5 × 10^3^ A/cm^2^ at room temperature (**g**,**i**,**k**) at the cathode, and (**h**,**j**,**l**) at the anode [31].

**Figure 15:**
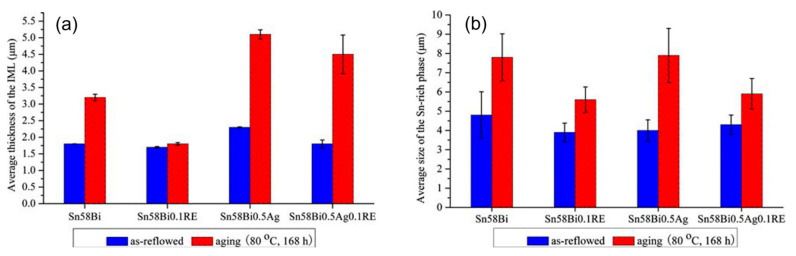
(**a**) Average thickness of intermetallic compound layer at the interface of the solder/Cu interface. (**b**) Sn–58 Bi metallurgical Sn phase average size [106].

In the study of adding rare earth elements to SnBi solder material, Hongwen He et al. [31] added 0.1% of rare earth element to SnBi solder material and conducted experiments at a current density of 5 × 10^3^ A/cm^2^ for a duration of 0–465 h. As shown in Figure 14, the samples without rare earth element exhibited cracks on the cathode and whiskers and hillocks extruded from the anode after prolonged electromigration tests. However, in the experiments with the addition of rare earth elements, the appearance of the solder joints after extended electromigration tests showed comparable IMC and Bi-rich layer formations to the control group without rare earth element. Dong et al. [106], in their investigation on the influence of trace rare earth additions on the microstructure and properties of Sn-Bi-based solder alloys, also demonstrated in Figure 15 that rare earth elements can inhibit the growth of IMC layers and intermetallic compounds at high temperatures. The results indicate that rare earth elements have a certain mitigating effect on electromigration [107].

### 4.2. Addition of Inorganic Filler into SnBi Solder

#### 4.2.1. Effect of Graphene Nanosheet Addition on the Properties of the Solder

Graphene nanosheets, when incorporated into SnBi solder, contribute to improved thermal and electrical conductivity, as well as enhanced mechanical strength. The two-dimensional structure of graphene nanosheets helps to hinder the diffusion of atoms and prevent electromigration-induced failures.

Graphene nanosheets (GNSs) are two-dimensional materials derived from graphene, with ultra-thin thickness, a melting point of 3652 °C, high strength and stiffness, excellent electronic structure, and high specific surface area [108]. When added to SnBi solder, GNSs significantly improve the mechanical properties of the solder and provide it with a certain degree of corrosion resistance [109]. Ma et al. [91] studied the effects of GNSs in Sn58Bi0.7Zn solder and found that GNSs act as dispersion and grain refinement agents that increase the load-carrying capacity of the solder. GNSs refine the grain structure throughout the solder joint. However, regarding atomic migration during electromigration, the effects of GNSs are contradictory. On one hand, GNSs provide more channels for atomic migration due to the refined grain structure. On the other hand, GNSs do not move under the current and thus block the migration of atoms undergoing electromigration [110].

**Figure 16:**
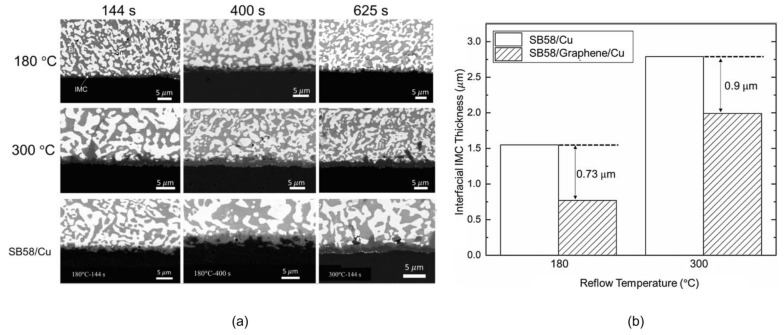
(**a**) Backscattered electron images of SB58/Graphene/Cu and SB58/Cu after the reflow processes at different reflow temperatures and times. (**b**) Interfacial IMC thicknesses in SB58/Cu and SB58/Graphene/Cu reflowed at 180 °C and 300 °C, respectively [112].

The grain refinement is due to the fact that GNSs are pinned at grain boundaries and inhibit the growth of grains [111]. It is worth noting that phase boundaries are considered to be the fast diffusion paths of Bi in SnBi alloys. Therefore, the finer the microstructure, the more diffusion channels are available for Bi migration toward the anode. Conversely, coarsening of the microstructure reduces the number of diffusion channels and suppresses the electromigration of Bi atoms. In addition, GNSs incorporated into the solder with high mechanical strength form rigid walls that inhibit the electromigration of Bi atoms [112]; this is demonstrated at temperatures of 180 °C and 300 °C in Figure 16 and the morphology shown in Figure 16a is accompanied by the data presented in Figure 16b. The addition of GNSs significantly reduces the Cu_6_Sn_5_ (IMC) thickness of the Cu base and weld interface, indicating that GNSs can effectively shield against the atomic diffusion process. Therefore, the role of GNSs in mitigating electromigration in SnBi solder depends on the experimental conditions.

#### 4.2.2. Effect of Fullerene Addition on the Properties of the Solder

Fullerenes exhibit unique properties that aid in reducing electromigration effects. Their spherical shape and high electrical conductivity allow them to effectively trap and neutralize migrating metal atoms, thereby impeding their movement and prolonging the lifespan of solder joints.

Fullerene (FNS) nanomaterial exhibits excellent electrical properties. Incorporating fullerene FNS in the study resulted in a reduction in the average thickness of the Cu_6_Sn_5_ (IMC) layer at the initial composite solder joint interface compared to conventional solder joints [113]. The FNS particles in the solder joint can act as barriers to inhibit the diffusion and migration of Sn and Cu atoms [114]. The addition of fullerene changes the chemical potential within the solder joint and alters the current density distribution, thereby modifying the atomic diffusion flux during electromigration and leading to a certain degree of thinning in the IMC thickness at both ends of the solder joint compared to joints without fullerene. Additionally, the inclusion of fullerene typically acts as an electron scattering center due to its non-conductive nature. When the volume fraction of fullerene in the solder joint is relatively high, the overall resistance of the joint noticeably increases [115].

Many academics also had to experiment with adding other organic materials. Zhang et al. [116] utilized cage-type polyhedral oligomeric silsesquioxane (POSS) trisilanol doping in their research on SnBi solder. They found that this material could delay electronic migration due to its electrically inert nature and its ability to react with metals. POSS trisilanol can slow down the occurrence of electronic migration and inhibits whisker formation [117]. Hu et al. [118] introduced graphene doping into Sn-8Zn-3Bi and discovered that this material could enhance microstructural and mechanical properties through a dispersion-strengthening mechanism [118], resulting in a well-controlled finer microstructure and the intrinsic excellent mechanical properties of graphene.

#### 4.2.3. Effect of Al_2_O_3_ Addition on the Properties of the Solder

The addition of Al_2_O_3_ in SnBi solder serves to alleviate electromigration to some extent. The primary effect of incorporating Al_2_O_3_ into the solder joint is a rapid increase in shear strength and microhardness. The secondary effects include retarding the growth of Cu_6_Sn_5_ (IMCs) and reducing the thickness of the bismuth-rich layer.

Al_2_O_3_ is relatively inert at room temperature and does not easily undergo reactions. It does not form other compounds when added to the SnBi solder system. Al_2_O_3_ has high thermal conductivity and insulation properties. The Al_2_O_3_ particles can to some extent impede the flux of Sn atoms from the anode to the cathode and Bi atoms from the cathode to the anode, resulting in a significant decrease in the diffusion coefficient of the overall solder compared to the control group [119]. Additionally, as particles act as barriers to hinder atomic diffusion, they further inhibit the growth of the IMC layer. Since SnBi solder is a low-temperature solder, it undergoes reflow after electromigration. However, the melting point of Al_2_O_3_ particles is relatively high, so the non-uniform nucleation of Al_2_O_3_ particles in the solder reduces the energy threshold for nucleation, promotes crystal refinement, and enhances the overall mechanical properties of the solder joint [120]. However, the addition of Al_2_O_3_ particles does not effectively solve the issue of IMC layer thickness growth. In the continuous generation of Joule heating during electromigration, the average thickness of the IMC will gradually increase regardless of the presence of Al_2_O_3_.

**Figure 17:**
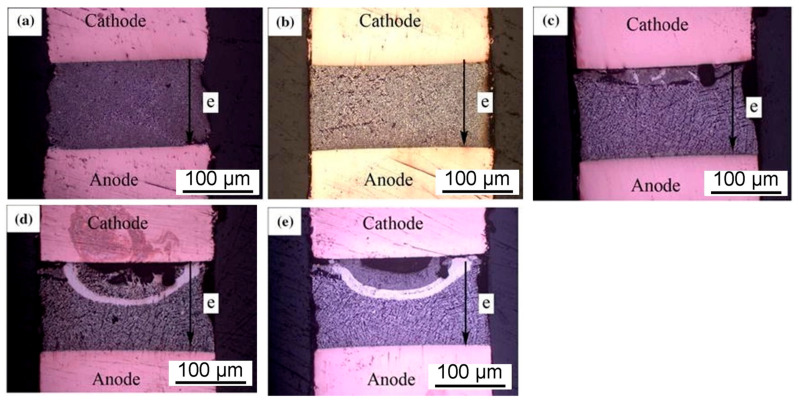
Macroscopic morphology of Cu/Sn-58Bi0.5Al_2_O_3_/Cu joints at different electromigration times: (**a**) 0 h, (**b**) 140 h, (**c**) 330 h, (**d**) 380 h, and (**e**) 410 h [121].

In the entire solder joint, the diffusion rate of Bi atoms under current stress is faster than that of Sn. Bi atoms, as the controlling diffusion components diffuse through Bi grain boundaries, Sn grain boundaries, and Sn/Bi interfaces. Scholars from Changshu Institute of Technology [121] conducted experiments to enhance the electromigration effectiveness of Sn–58Bi solder. In the experiment, Al_2_O_3_ was added at a proportion of 0.5% of the total amount, and it was found that Al_2_O_3_ played a certain inhibitory role in electromigration. Metallographic analysis revealed the formation of white stripes in the solder joint, as shown in Figure 17. The presence of Al_2_O_3_ successfully slowed down crack propagation and diffusion. The main component in the white arc detected during the electromigration experiment in the experimental group was Bi.

As can be seen from the results, the thickness of the IMC was significantly smaller in the group with Al_2_O_3_ compared to the control group without Al_2_O_3_. From a morphological perspective, it can be concluded that the addition of Al_2_O_3_ particles effectively alleviated the atomic migration during electromigration.

### 4.3. Other Improvement Options

Without altering the composition conditions, modifying the crystal orientation within the solder joint or introducing stress into the joint can improve its reliability to a certain extent.

One way to avoid adding modified reinforcement phases to solder is to make some changes to the crystal orientation in the solder or to do so before electromigration [122]. When the β-Sn crystal c-axis and current form a certain angle θ, a it was observed that high θ angle reduces the Cu migration and lower θ angle facilitates the Cu atoms migration which results in EM failures. When the direction of the current and the direction of the c-axis are in the same direction, the number of atomic migrations and defects caused by electromigration increases rapidly [123].

Copper atoms diffuse into rapidly diffusing grains and form along the tilted twin boundaries, which are low-energy and highly correlated [124]. However, interestingly, Cu_6_Sn_5_ grows rapidly in areas with lower copper diffusion rates. The outline of the Cu_6_Sn_5_ (IMC) follows the high-angle grain boundary between the fast and slow diffusing grains. This grain boundary IMC is the result of Cu diffusion through fast diffusing grains, but it implies that both the growth of IMC in the solder bulk and along the grain boundaries occur in the fast-diffusing grains since it contacts the cathodic interface, i.e., the source of copper atoms in the electromigration. The probability of defect generation due to electromigration increases rapidly when the angle between the Sn crystal and current direction is less than 45°. The growth of IMC in the solder joint is a direct outcome of copper diffusion from the Cu substrate, driven by the rapid and effective diffusion rate in large, low-angle grains. The planar growth of IMC along the grain boundaries is the result of fast diffusing grains, slow diffusing grains, and grain boundary diffusion.

In addition, there are significant differences in the accumulation of Cu dissolution and IMC formation in β-Sn under “forward” and “reverse” current stresses [125]. For instance, when electrons flow in the “forward” direction, electromagnetic induction causes a large amount of Cu to diffuse towards the anode due to the small angle of the β-Sn grains, resulting in severe dissolution of Cu at the cathode. However, diffusion of Cu on grain boundaries is significantly delayed due to the large angle between neighboring β-Sn grains, leading to the accumulation of Cu atoms at grain boundaries and the precipitation of columnar Cu_6_Sn_5_ grains inside β-Sn grains. On the other hand, when electrons flow in the “reverse” direction, the diffusion flux of Cu induced by EM towards the anode is reduced due to the larger angle of β-Sn particles, which limits the dissolution of Cu at the cathode. Since the angle between neighboring b-Sn grains is smaller, the diffusion of Cu on grain boundaries is not hindered. As a result, Cu atoms diffuse to the anodic interface and precipitate as interface Cu_6_Sn_5_.

When pre-machining occurs before electromigration, the cavities within the material merge into a single large void. The dislocations resulting from plastic pre-strain initially act as sinks for vacancies during the electromigration process. However, as vacancies accumulate at these dislocations, their climb leads to the recovery of the deformed sample under the current stress, thereby reducing the density of dislocations and vacancies in the solder. This results in a slower diffusion of Bi atoms.

Zhang et al. [126] researchers applied pre-stress to the SnBi filler metal prior to electromigration to increase the atomic vacancies within the filler metal. They tested various levels of strain, namely 0%, 2%, 8%, and 20%, respectively. The experiments utilized a current density of 1.3 × 10^4^ A/cm^2^, with an electromigration duration of 180 h. The findings revealed that higher levels of strain applied during the experiment led to a thinner Bi-enriched layer formed at the anode after electromigration. An intriguing observation emerged from this experiment: contrary to previous theories suggesting that greater defects result in larger atomic flux during electromigration, the sample with the highest level of strain exhibited the smallest Bi-rich layer at the anode. These results indicate that deformation increases the presence of dislocations and other defects, which may not uniformly align with the direction of electromigration. Additionally, the climb and glide of dislocations within the original solder are restored, acting as more effective sinks for vacancies and reducing vacancy density. This process slows down the diffusion rate of Bi atoms. Introducing pre-strain presents a potential solution to enhance the electromigration resistance of solder interconnects by mitigating electromigration-induced damage.

## 5. Discussion on the Mitigation Effects of Electromigration among Different Methods and Measures

The majority of the metallic additives discussed in this paper form compounds within the solder joints. These intermetallic compounds are not affected by the current and remain stable during atomic migration, acting as barriers to prevent atomic diffusion. Additionally, interesting findings were observed when investigating rare earth and Zn elements. The addition of rare earth elements results in grain refinement and improved mechanical properties within the solder joint. However, the increased number of interfaces leads to an increase in the number of transmission channels, so the addition of rare earth elements does not necessarily enhance reliability. Silver (Ag) exhibits similar behavior within the solder joint. On the other hand, zinc (Zn) migrates in the opposite direction of atomic migration within the solder joint, counteracting momentum and thereby enhancing the reliability of the joint.

Non-conductive inorganic materials, relying on their own properties, can stably exist within solder joints and play a role in improving reliability. However, due to their high electrical resistivity, they contribute to the overall resistance of the solder joint. During the process of electromigration, the rapid increase in temperature accelerates the failure of the solder joint. When conductive inorganic materials are added, the effect of refining the grain structure can rapidly improve the mechanical properties of the solder joint. However, similar to the addition of rare earth elements, they provide a large number of channels that accelerate atomic migration.

There are few studies on improving solder joint reliability without the addition of other substances. The crystal orientation of the material itself changes under the continuous influence of current during electromigration. It is worth noting that the introduction of stress increases the number of defects within the solder joint, leading to an increase in resistance, temperature rise during electromigration, and remelting of the joint. As a result, the stress and voids within the joint redistribute, making it suitable only for non-molten state applications. The Table 1 and Table 2 below summarize the approaches and methods discussed in this paper for enhancing mechanical performance. From an economic perspective, delaying electromigration by using additives is the most cost-effective option compared to not using any additives. However, this approach has high relative process requirements, leading to increased costs in the process. Rare earth (RE) is more expensive than graphene nanosheets (GNSs) and fullerene nanosheets (FNSs), which are in turn more expensive than other metals. Among these, the least expensive metal currently is iron (Fe). Each of the methods discussed in this article may have additional effects on the process of delaying electromigration, which could prove to be economically beneficial in various scenarios.

## 6. Conclusions

Advanced packaging technologies have facilitated the development of electronic components towards miniaturization, high performance, and multifunctionality. As the size of solder joint decreases, issues related to electromigration (EM) and thermomigration (TM) become increasingly severe. This paper provides an overview of the principles behind electromigration, which involve the transfer of momentum from electrons to atoms, causing atomic movement in the direction of current flow. When the force exerted by electron wind is weaker than the force exerted by concentration gradients, metal elements diffuse into the solder matrix and eventually lead to phase separation. Electromigration during this process can result in various problems such as changes in solder joint morphology, crack formation, phase separation, and a significant increase in resistance. The methods for improving the reliability of solder joints during electromigration are summarized and discussed, focusing on the following aspects:

1. Adding metal elements to form intermetallic compound in SnBi solders can effectively alleviate the phenomenon of atomic migration during electromigration. Metals that can form compounds with the solder or the substrate are first considered. These compounds can effectively resist atomic migration. Fe and Sn form FeSn_2_, which is dispersed in the solder and Cu_6_Sn_5_ layer to hinder electromigration. This compound prevents the problem of excessive growth at high temperatures after electromigration. Ni and Sn can form intermetallic compounds such as (Ni_1−x_Cu_x_)_3_Sn_4_, Ni_7_Sn_2_Bi, NiBi, and NiBi_3_. Ni atoms replace Cu atoms in Cu_6_Sn_5_ to form (Cu,Ni)_6_Sn_5_, which is more stable than Cu_6_Sn_5_. Therefore, it can alleviate the defects caused by electromigration. Ag forms Ag_3_Sn in the solder and has unparalleled stability under all soldering conditions. During solid-state diffusion, concentration gradients and high temperatures induce interactions between the solder joint and the Cu substrate. This compound completely changes the structure of the solder joint after electromigration. Co in the solder also forms compounds that inhibit electromigration. It should be noted that the mechanism by which Zn enhances reliability in the solder joint is different from that of other metal elements. Zn itself undergoes reverse migration, thereby hindering atomic migration.
2. Adding rare earth materials and inorganic filler materials to SnBi solders can improve the reliability of solder joints based on their own properties. Adding rare earth materials to SnBi can refine the grain size and gather at the grain boundary to inhibit slip or climb of dislocations, reducing defects. Graphene nanosheets and fullerenes have unique characteristics that enhance reliability. Graphene nanosheets have a high melting point and form rigid barriers to prevent atomic migration. Fullerenes, while not having a high melting point, can alter the chemical composition within the solder, thereby impeding the generation of Kirkendall voids. As a non-conductive material, Al_2_O_3_ can be added to the SnBi solder, which can reduce the atomic flux during electromigration. The diffusion coefficients of elements in the solder are reduced. At the same time, Al_2_O_3_ particles act as barriers to hinder atomic diffusion and further suppress the growth of the Cu_6_Sn_5_ layer.
3. Without adding other substances, stress engineering can be considered from the aspects of crystal orientation and pre-strain before current flow. When the crystal orientation and current direction form a certain angle, a portion of the electron wind will deflect the crystal. A critical θ angle exists between the c-axis of the crystal and the current direction. When the angle between the current and crystal orientation is lower than θ angle, the atomic migration and defect density caused by electromigration will increase rapidly. Dislocations induced by plastic pre-strain play the role of vacancy sinks in the early stage of electromigration. However, with the accumulation of vacancies at dislocation sites, the climbing of these dislocations promotes the recovery of deformation samples under current stress, greatly reducing the dislocation and vacancy density in the solder, resulting in slower diffusion of Bi atoms.

## Figures and Tables

**Table 1:** Element and filler addition method in the solder.

Additive	Solder	Methods for Improving Electromigration	Reference
Fe	SnBi	FeSn_2_, Fe_2_O_3_ hinder atomic diffusion	[56,57,58,59,60]
Ni	SnBi	The formation of compound (Cu, Ni)_6_Sn_5_ hinders atomic migration	[68,69,70,71,72,73]
Ag	SnBi	The formation of compound Ag_3_Sn obstructs atomic migration and refines grain	[79,80,81,82,83,84,85,86,87,88]
Zn	SnBi	Zn diffuses in the opposite direction to the main migratory material, hindering migration	[92,93,94]
Co	SnBi	Form a large amount of (Co, Cu)Sn_2_, barrier material migration	[96,97]
RE	SnBi	Rare earth elements accumulate towards the grain boundaries, blocking the transport of atoms	[103,104,105,106,107]
GNSs	SnBi	GNSs blocks atomic migration	[111,112]
FNSs	SnAgCu	The FNSs particles become a barrier, preventing atomic migration	[113,114,115]
Al_2_O_3_	SnBi	Al_2_O_3_ prevents atomic migration	[116,117,118,119,120,121]

**Table 2:** Addition-free method in the solder.

Improved Technology	Solder	Methods for Improving Electromigration	Reference
Orientation change	SnBi	Large grain boundaries hinder the diffusion of atoms	[122,123,124,125]
Stress application	SnBi	Add stress to increase the vacancy in the weld and reduce the migration speed of Bi	[126]
